# Does Osteopathic Manipulative Treatment Induce Autonomic Changes in Healthy Participants? A Thermal Imaging Study

**DOI:** 10.3389/fnins.2020.00887

**Published:** 2020-08-18

**Authors:** Francesco Cerritelli, Daniela Cardone, Alessio Pirino, Arcangelo Merla, Fabio Scoppa

**Affiliations:** ^1^Clinical-based Human Research Department, Foundation COME Collaboration, Pescara, Italy; ^2^Department of Neuroscience and Imaging, Institute for Advanced Biomedical Technologies, University G. D’Annunzio of Chieti-Pescara, Chieti, Italy; ^3^Department of Biomedical Sciences, University of Sassari, Sassari, Italy; ^4^Faculty of Medicine and Dental Surgery, Sapienza University of Rome, Rome, Italy; ^5^Chinesis I.F.O.P. Osteopathy School, Rome, Italy

**Keywords:** touch, autonomic nervous system, cholinergic system, sham, hrv, gsr

## Abstract

Osteopathic manipulative treatment (OMT) has been demonstrated to be an effective therapy in several clinical conditions and age groups. Despite the clinical effectiveness, lack of robust data in terms of neurobiological, specifically autonomic, mechanisms of action is observed. Preliminary studies showed a parasympathetic effect leading to a trophotropic effect of OMT. However, these data are limited to heart rate variability (HRV) analysis. In order to study further the role of OMT on the autonomic nervous system, a cross-over randomized controlled trial RCT has been designed to test the effect of osteopathic treatment compared to sham therapy on a range of autonomic parameters. Thermal images, HRV and skin conductance data were collected on a sample of healthy adults. The study design consisted of two sessions (OMT and SHAM), 1 treatment per week, lasting 35 min each, composed of 5 min of baseline, 25 min of treatment, and 5 min of post-touch. During the baseline and the post-treatment, participants received no touch. Thirty-seven participants (aged 27 ± 5 years old, male ratio 40%) completed the study. Multivariate analysis showed a significant parasympathetic effect of group as well as of epoch on thermographic data of the nose (estimate 0.38; 95% CI 0.12–0.63; *p* < 0.01), left (0.17; 0.06–0.27; <0.001) and right (0.16; 0.07–0.24; <0.001) perioral as well as on the forehead (0.07; 0.01–0.12; <0.01) regions but not for the chin (0.08; −0.02 to 0.18; 0.13). Consistent with a parasympathetic effect, analyses demonstrated a difference between OMT and sham groups on the nuHF (*p* < 0.001) and DFA-a1 (*p* < 0.01) as well as on skin conductance (<0.01). The present research supports the hypothesis that a single session of OMT as compared to sham induces autonomic consequences in healthy non-symptomatic adults. Clinicaltrial.gov identifier: NCT03888456, https://clinicaltrials.gov/ct2/show/NCT03888456.

## Introduction

Osteopathy is a complementary touch-based manual medicine primarily used by different age groups of patients (Licciardone et al., 2013; Cerritelli et al., 2014, 2015b; Pizzolorusso et al., 2014; Luciani et al., 2015, 2018; Schwerla et al., 2015; Steel et al., 2017) and in a wide variety of clinical conditions (Cerritelli et al., 2011, 2015a, 2016a, 2017b; Cicchitti et al., 2015; Ruffini et al., 2016; Franke et al., 2017; Lanaro et al., 2017). Several studies showed positive clinical effects as compared to sham, placebo, usual care or physical devices (Pizzolorusso et al., 2011; Cerritelli et al., 2013, 2015a, 2016b; Martelli et al., 2014). Notwithstanding, however, the reported clinical effectiveness of osteopathy from several studies (Cerritelli et al., 2019), the underpinning mechanisms of action of osteopathic manipulative treatment (OMT) remains an open question to explore further. And yet, this is crucial to tackle in order to identify, more precisely, the neurobiological pathways of the OMT, which can be of help for future clinical and research applications.

Based on the current evidence, it is difficult to identify what are the main neurobiological elements involved during and after administering an osteopathic session. Indeed, it has been suggested that possible OMT mechanisms are modulated by the autonomic nervous system (ANS) functions (Ruffini et al., 2015; D’alessandro et al., 2016), where interoceptive mechanisms might be involved (Cerritelli et al., 2020), leading to a reduction of pro-inflammatory cytokine release (Licciardone et al., 2012; Degenhardt et al., 2017). This evidence was shown both *in vitro* (Zein-Hammoud and Standley, 2015) and *in vivo* (Degenhardt et al., 2017), suggesting an anti-inflammatory role of OMT (D’alessandro et al., 2016). McGlone et al. (2017) argued that OMT could putatively decrease cytokine production and sympathetic activity, generating a cascade of physiological and neurobiological events, which in turn can modulate the inflammation and the ANS reactivity.

The activity of the sympathetic and parasympathetic systems might play a central role in the OMT effects. Ruffini et al. (2015) demonstrated on healthy adults that one session of OMT, compared to sham and no intervention, induces immediate parasympathetic effects suggesting a trophotropic effect. The authors measured autonomic response using linear and non-linear parameters derived from heart rate variability (HRV) while participants laid supine in a temperature-humidity controlled room. Fornari et al. showed in a more recent study, enrolling healthy subjects, who underwent a laboratory stress episode, i.e., mental stressor, that OMT as compared to sham therapy produced a chronotropic effect (reducing the heart rate) and an induced sympathovagal balance (Fornari et al., 2017), thus proposing a crucial role of ANS in the context of the action of osteopathy. However, it is worth to note that the majority of scientific studies, investigating the role of ANS in osteopathy, focused their attention mainly on one of the autonomic parameters, the HRV, showing higher high frequency (HF) values, lower low frequency (LF) values as well as lower LF/HF ratio. Therefore, it could represent a limited measure as compared to the variety of physiological mechanisms controlled by the ANS. To further understand the mechanisms, it would be necessary to combine different autonomic measures. The current study is designed to meet this need employing thermal InfraRed Imaging (IRI) to assess regional facial temperature, and simultaneous measurement of cardiac and skin activity to derive autonomic parameters such as electrodermal activity (EDA) and HRV.

In this regard, IRI allows researchers to precisely estimate cutaneous temperature, a proxy of autonomic activity, through a contactless technique. The validity of IRI has been extensively demonstrated by studies showing how autonomic activities can be depicted from specific facial temperature patterns induced by different physical or psychological states (Shastri et al., 2009; Ioannou et al., 2014; Merla, 2014; Cardone and Merla, 2017). The specific variability of temperature patterns in distinct, standardized facial regions has been regarded as reflecting the activation or deactivation of the sympathetic nervous system. For instance, an increase of the temperature in the periorbital regions is reported to be a proxy for a flight or fight response, while nasal skin temperature variation represents a specific autonomic effect (Ioannou et al., 2014). In particular, for the nasal area, an increase of temperature to a baseline condition is indicative of a parasympathetic activation while a decrease of temperature implies a sympathetic effect (Cardone and Merla, 2017). Moreover, the reliability of IRI as a tool for the detection of the psycho-physiological state of the participants has been proven by simultaneous EDA recordings, more specifically, galvanic skin response (GSR) measurements. Galvanic skin response signals have been shown to correlate with the number of active sweat glands, which activation can be easily revealed by facial thermal IR imaging by the appearance of cold dots over the face. Together with the hands’ palm area, strong activation of the sweat gland is shown in the maxillary, perioral, and nose tip regions.

Multiresolution analysis of the temperature signals reveals tonic (baseline and/or general) and phasic (event-related) components strongly correlated with GSR sympathetic constituents (Shastri et al., 2009; Krzywicki et al., 2014). Thus, examining changes in regional temperature over time has been considered a suitable option to study the ANS additionally.

In this context, only one study has been carried out in osteopathy, using thermal imaging. Polidori et al. (2018) recently published a proof-of-concept study, using a case report, which tested the use of IRI as an additional diagnostic tool in the osteopathic procedure. The authors showed that IRI thermography was able to detect low back pain changes immediately after OMT. However, as pointed out by the authors, this exploratory research, with all the limitations of the case study, might pave the way for further trials to measure the temporal effect of OMT using IRI accurately. The present study, therefore, aims at using a combination of measures including IRI, GSR, and HRV to investigate autonomic correlates of changes from baseline levels of healthy participants during and after OMT. The hypothesis is that the OMT, as compared to the control condition, would induce a more robust parasympathetic response.

## Materials and Methods

The experimental protocol was designed for a randomized-controlled single-blinded cross-over study. It enrolled 37 healthy participants, of either gender (*M* = 19; 40%), aged between 18 and 35 years old (27.2 ± 5.1), and who did not undertake any pharmacological treatment during the previous 4 weeks and were naïve to osteopathic treatment. Exclusion criteria were intended to avoid any condition that might have any significant autonomic effect and included: any cardiovascular, neurological, musculoskeletal, psychiatric, genetic or congenital disorders, current pregnancy or breastfeeding, and menstrual flow during the session. Smokers, as well as drug abuse participants, were excluded. Participants were asked to refrain from alcohol, caffeine and cardiovascular exercise for 24 h prior to the experimental session to control for external confounders.

Volunteers from different universities were recruited from March 2019 to May 2019 through e-mail, phone, or direct contact. Participation in the study was volunteered, and no reimbursement was provided to the participants.

The Institutional Ethics Committee of the University, “G. D’Annunzio” of Chieti-Pescara, approved the study and written informed consent from all participants were obtained before the experiment according to the Declaration of Helsinki. The trial was registered on clinicaltrials.gov identifier: NCT03888456.

### Randomization

**FIGURE 1:**
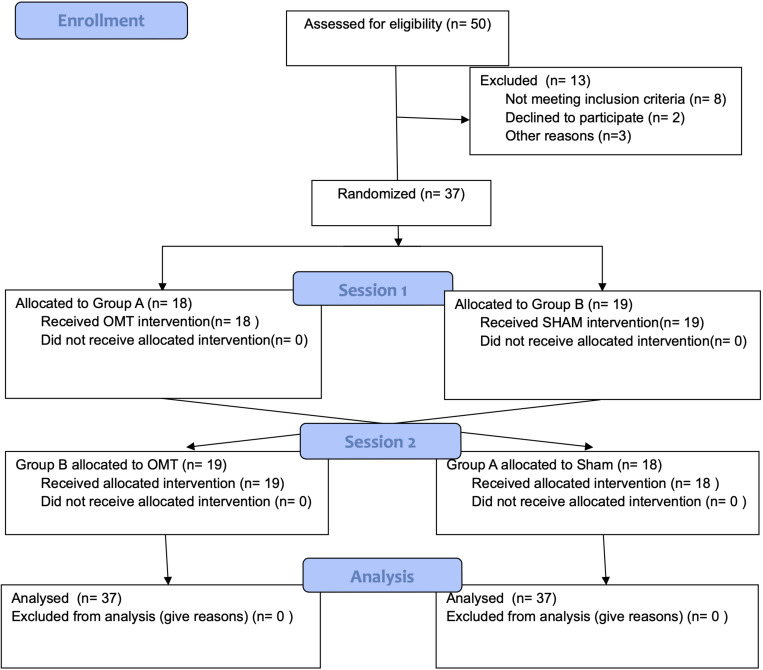
Flow-chart of the study.

Participants underwent a thermal imaging protocol and were randomly divided into two groups using a 1:1 ratio and were assigned in the first session to either the OMT (Group A) or the SHAM group (Group B) (Figure 1). Block randomization was performed according to a computer-generated randomization list using a block size of 9. Participants were unaware of the study design and outcomes as well as of group allocation. The randomization was performed and stored in a secure web-based space and an information technology consultant was responsible for the process.

### Baseline Assessment

Before the thermography session, participants were asked to complete paper-based questionnaires. The socio-demographic questionnaire was administered to collect baseline data in terms of age, gender, BMI, marital status, academic degree, type of work. The State-Trait Anxiety Inventory (STAI-Y1 and Y2) was used to test trait anxiety (Spielberger et al., 1983) and the Edinburgh Handedness Inventory was utilized to investigate the hand dominance (Oldfield, 1971).

### Experimental Design

The study design consisted of two sessions over 2 weeks, 1 session per week, lasting 60 min each, composed of 20 min rest period before measurement, 5 min of baseline, 25 min of treatment, 5 min of post-touch and the last 5 min to fill the post-session questionnaires. During the baseline and the post-treatment, participants received no touch.

The first session could be rescheduled if the participant presented at least one of the following exclusion criteria: acute pain in the last 72 h, consumption of any medicine or drug in the 72 h before the session, menstrual flow during the intervention day, alcohol consumption in the last 48 h. Participants were considered drop-out in case of non-attendance at the second session. Participants allocated to treatment groups received both OMT and sham therapy session at different time points based on the cross-over design. At the first session, group A received OMT while group B received sham treatment. Subsequently, in the second session, group A received sham treatment while group B received OMT.

Manual sessions were performed in the same room, with stable temperature and humidity, to avoid any influence of bodily thermoregulation (Cardone and Merla, 2017). Moreover, the two sessions were scheduled at the same hour to account effects due to the circadian rhythm. The OMT intervention consisted of an initial manual assessment followed by a treatment. The evaluation aimed at localizing somatic dysfunctions according to tissue alteration, asymmetry, range of motion and tenderness parameters (TART) which led the osteopathic evaluation and treatment (Cerritelli et al., 2017a). Somatic dysfunctions were found in the whole body, then balanced one by one to identify a primary order of intervention based on TART parameters. Osteopathic manipulative treatment techniques were focused on correcting the somatic dysfunctions found during the initial physical examination and included balance ligamentous techniques, balance membranous techniques, and cranio-sacral techniques (Magoun, 1976). All techniques that required contact with the head were excluded. For this trial, sham therapy mimicked, without applying any technique, the procedures used for osteopathic care. The osteopathic session lasted 25 ± 1 min. The sham procedure entirely overlapped the OMT in terms of contact time, session length, treatment context. Specifically, the sham session consisted of a gentle static touch applied, using the palm of the hands, over the 25 min among the following areas: upper and lower limb, pelvis, abdomen, thorax, and vertebral spine. Each area was touched between 3 and 4 min without paying attention to the area contacted. The operator was engaged in an auditory endogenous covert oriented attention task (Cerritelli et al., 2017a) and randomly chosen the sequence of bodily areas to touch.

All interventions, both OMT and sham, were performed by the same operator (man, 40 years old with 15 years of osteopathic clinical experience, certified osteopath DO trained following the WHO benchmark). Besides, the participants were asked to lie still and keep the eyes closed during the baseline, treatment, and post-touch periods.

### Post-session Rating

Several measurements were acquired at the end of the experimental session in order to assess the quality of the received touch. The Touch Perception Task (Guest et al., 2011) was used to describe the type of touch perceived by the participants during the sessions. In addition, a 5-point Likert scale was administered to classify the touch received by participants (1 = very light, 2 = light, 3 = moderate, 4 = heavy, 5 = very heavy). Moreover, the participants were invited to complete the Amsterdam Resting State Questionnaire to report the perception of their feeling during the session (Diaz et al., 2013).

### Procedure

Prior to testing, each participant was left in the experimental room for 20 min to allow the baseline skin temperature to stabilize (Cardone and Merla, 2017). As per standard guidelines in IRI research, the recording room was set as a thermoneutral environment, i.e., at standardized temperature (23°C) and humidity (50–60%) controlled by a thermostat, to avoid thermoregulatory-induced alterations. Participants sat comfortably on a chair during the acclimatization period, while during the measurement period participants laid down on a treatment table.

### Data Recording and Preprocessing

#### Thermal Imaging

The facial temperature was recorded using a digital thermal infrared camera FLIR SC660 (FLIR, Wilsonville, OR, United States) (640 × 480 bolometer FPA, noise equivalent difference temperature (NEDT): <30 mK @ 30°C, field of view: 24° × 18°).

The camera was placed 60 cm above the participant and pointed toward the face of the participant. The sampling frequency was 10 Hz. To remove the effects related to the potential drift/shift of the sensor’s response and optical artifacts, the camera was blackbody-calibrated. The quality of recorded IRI was checked by visual inspection. No videos were excluded.

According to the literature, perioral, nasal tip, chin, and forehead areas were chosen as regions of interest (ROIs; Garbey et al., 2007; Shastri et al., 2009; Ioannou et al., 2014). The following criteria were used to ensure a reliable positioning and sizing of the ROI: for the nasal tip, a circular ROI was placed over the nasal center and did not extend beyond the nostrils; for the perioral regions two ellipses whose longer axis ran from nose to mouth boundaries, and shorter axis was half of the longer one; for the chin, a circular ROI was placed on the mental protuberance; for the forehead, a circular ROI was positioned in the center of the midline frontal region. Within each IRI sample image, these ROIs moved together with the relative movement of the face employing; therefore, a soft-tissue tracking algorithm, validated in several research works (Manini et al., 2013). When the tracking algorithm failed (e.g., because of too much head rotation, a deep variation of thermal pattern for the first initializing frame), the failure was displayed as a large variance of the extracted signal, which was corrected by visual inspection by substituting contaminated samples with the mean value of six samples before and after the period. The average artifact-corrected temperature within the selected ROI was considered indicative of autonomic activity (Cardone and Merla, 2017).

The IRI signals were filtered with a zero-lag third-order Butterworth low-pass filter (0.4 Hz) to eliminate the high-frequency oscillations that were unrelated to autonomic modulations (Pinti et al., 2015).

Each participant’s time series was then z-transformed (subtracted by their average value and divided by their standard deviation) (Rosenblatt, 1952) to account for between-participant variance in IRI amplitude.

#### Skin Conductance

Skin conductance response (SCR) was recorded on the thenar/hypothenar muscles of the non-dominant hand (Ogorevc et al., 2013) using the AD instrument Powerlab system, which provided a GSR amplifier with low voltage, 75-Hz AC excitation, and automatic zeroing. The finger electrodes were made by stainless steel and were held with Velcro tape. The sampling frequency was 1 kHz. The SCR signal was filtered with a zero-lag third-order Butterworth bandpass filter (0.01–5 Hz) (Bach et al., 2010) and then down-sampled to 10 Hz to be homogenized with the IRI. The tonic and phasic components of the signal were separated using a continuous decomposition analysis provided by Ledalab, which is a Matlab-based software (Benedek and Kaernbach, 2010). The SCR signal was then z-transformed.

#### HRV Data

Cardiac signals were recorded by means of a finger pulse transducer, using the AD instrument Powerlab system. A finger pulse transducer is a piezo-electric element, able to convert the mechanic force applied to its active surface into an analog electrical signal. Similarly, to what is done to the ECG signal in terms of HRV calculation, it is possible to extract the pulse rate variability (PRV) from the finger pulse signal. As PRV can be used as a surrogate for HRV at a resting position (Shin, 2016), the analogous of the R–R intervals were extracted from the finger pulse signal. Using customized software, outliers were identified and removed from the data. Intervals were then imported in Kubios software^1^ to compute HRV parameters.

HRV analysis method, based on processing recorded RR intervals, was divided into linear analysis (time and frequency domain) and non-linear analysis (Aubert et al., 2003). From power spectra (Fast Fourier transformation using Blackman Harris window) of equidistant linear interpolated (4 Hz) tachograms (resampled to 2 Hz), the following frequency domain standard HRV index was used for linear analysis: nuHF, from 0.15 to 0.4 Hz, i.e., signal of parasympathetic heart rate modulation (No authors listed, 1996). Considering non-linear analysis, the DFAα1 parameter was computed. DFAα1 is considered a sensitive parasympathetic index (Kemp et al., 2012) able to discriminate possible long-term correlations and complexity of RR interval series (Hardstone et al., 2012). A fractal structure of heart rate was quantified by estimating a short-term, alpha 1, fluctuations, obtained from the range 4 ≤ *n* ≤ 16.

### Data Analysis

#### Thermal Data Analysis

On the participant level, thermal data were averaged into 30-s-long time bins and subdivided into baseline, touch and post-touch periods. Due to the nature of thermal data, that is sensitive to movement artifacts, and to avoid non-experimental movements, individual participant’s baseline values containing data that was larger or smaller than three SDs of the whole sample mean were selected and considered to be artifacts. Such data were then replaced by the participant’s non-artifactual epochs mean from the Baseline period. Afterward, each 30-s long data from the Touch and Post-touch periods were converted to a change from Baseline, by subtracting that participant’s mean baseline from each of the epochs in the Touch and Post-Touch periods. Again, for each participant, data points which lay more than three SDs outside the grand mean for the sample were identified and artifactual epochs were replaced with the mean of that participant’s non-artifactual epochs within a given time period.

#### Skin Conductance Analysis

Skin conductance response was analyzed following the same procedure used for thermal data. Indeed, SCR data were split, and averaged into 30-s bins and broke down into baseline, touch, and post-touch period. Then artifacts were identified and corrected.

#### HRV Analysis

As HRV analysis is considered, the restricted weak stationarity (RWS) test was performed to assess stationarity (Magagnin et al., 2011) over *M* patterns. To test the normality of R–R distribution (*p* < 0.05), the Kolmogorov Smirnov goodness-of-fit was used. In case of non-normal distribution, a log transformation was applied. Subsequently, *M* patterns were tested for normality. The patterns were randomly chosen from a set of sequences of length *L* (Magagnin et al., 2011).

The arithmetic mean and standard deviation, as well as median, percentage, and range were used to explore the general characteristics of the study population.

To compare the OMT and sham group at enrollment, univariate statistical tests, student *t*-test and chi-square test were performed. To study the independent effect of OMT on thermic, skin response and HRV endpoints, a repeated measure analysis based on mixed-effect regression (MER) model considering random effect for groups and a fixed effect for period was used to explore any difference further. *Post-hoc* pairwise analysis adjusted by Holm-Bonferroni correction was utilized after any statistical difference resulted from MER.

### Statistical Analysis

Before the investigation, the number of participants (n) was calculated. The neuroscience literature in thermography reported expected effect size estimates to be relatively high. Choosing, therefore, an effect size of 0.7, together with an alpha value of 0.05 and a Beta of 0.80 typical in clinical studies have been included in the R statistical program to estimate the sample size. Intending to reveal, also, intraindividual as well as interindividual differences, a total of *n* = 35 persons were examined in a crossover design.

To indicate statistical difference, two-tailed *p* values of less than 0.05 was considered. The effect size was computed using Cohen’s d. This data analysis was carried out using *nlme*, *multcomp, stats, effsize* packages of the R statistical program (v. 3.5.2).

## Results

### Sample Characteristics and Baseline Measurements

Fifty participants were screened, of whom 37 met the entry criteria, gave written informed consent and were randomly assigned to the study intervention groups (Figure 1). The demographic characteristics and baseline values are shown in Tables 1, 2 showing no statistically difference at the baseline among groups.

**TABLE 1:** Demographic and clinical characteristics of the OMT and sham group at baseline.

Characteristics	OMT	Sham	*p* > |*t*|
**Demographic**			
Age	27.1 ± 5.0	27.6±5.3	0.76
Female sex (%)*	13 (68)	10 (56)	0.64
BMI	22.3 ± 4.6	23.4 ± 3.3	0.40
Civil state (%)*			1.00
Not-married	17 (89)	17 (94)	
Married	2 (11)	1 (6)	
Education title (%)*			0.58
High school	11 (58)	11 (61)	
Academic degree	8 (42)	7 (39)	
**Clinical**			
STAI – Y1	43.3 ± 4.2	45.1 ± 4.3	0.14
STAI – Y2	43.0 ± 4.5	42.5 ± 5.2	0.76

Data are presented as mean ± standard deviation. *N(%). *p* Values from student *t* test. *from X^2^ test. BMI, Body Mass Index; STAI, State-Trait Anxiety Inventory.

**TABLE 2:** Thermic and galvanic skin response baseline data.

Characteristics	OMT	Sham	*p* > |*t*|
Thermic			
Forehead	36.5 ± 0.7	36.3 ± 0.7	0.43
Nose	34.5 ± 3.3	34.4 ± 3.8	0.92
Right Perioral	36.0 ± 1.3	35.9 ± 1.2	0.76
Left Perioral	35.9 ± 1.6	35.8 ± 1.5	0.80
Chin	35.9 ± 1.2	35.7 ± 1.2	0.67
GSR	1.1 ± 3.4	2.5 ± 2.3	0.10
Heart rate	69.9 ± 3.7	69.8 ± 4.1	0.92

*Data are presented as mean ± standard deviation. *p* values from student *t* test. GSR, galvanic skin response.*

Besides, there were no differences in terms of type of touch felt between sessions (session 1: mean 8.15 ± 1.8; session 2: 8.36 ± 1.4; *t* = -0.56, df = 69.7, *p*-value = 0.58) and the type of touch received across sessions (*X*^2^ = 0.01, *p*-value = 0.99).

### Temperature Changes

**FIGURE 2:**
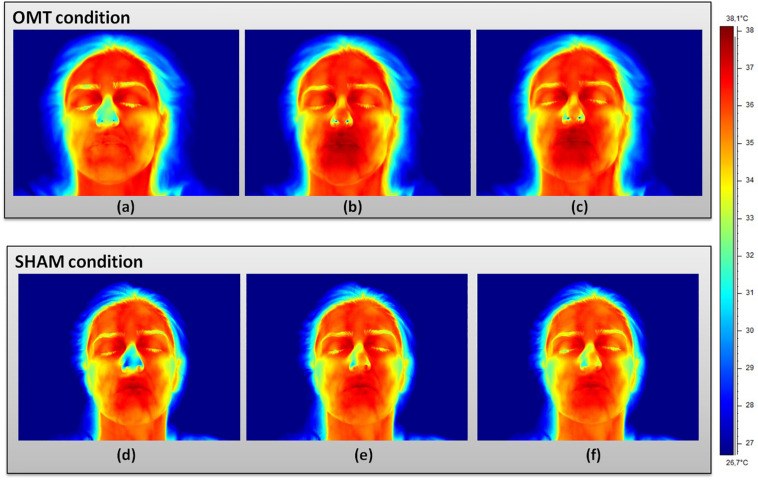
Facial thermal changes in a representative participant receiving either osteopathic manipulative treatment or sham. Panels **(a,d)** represents baseline period before the touch timulation. Panels **(b,e)** show touch period. Panels **(c,f)** demonstrate post-touch period. A general temperature increase can be observed over the whole face in the osteopathic group as compared to the sham. In particular, while the chin slightly changes their average temperature values, nose tip, perioral, maxillary, and forehead regions clearly present a temperature increase where red areas can be easily spotted. OMT, osteopathic manipulative treatment.

Figure 2 showed an example of thermic images for the two groups.

**FIGURE 3:**
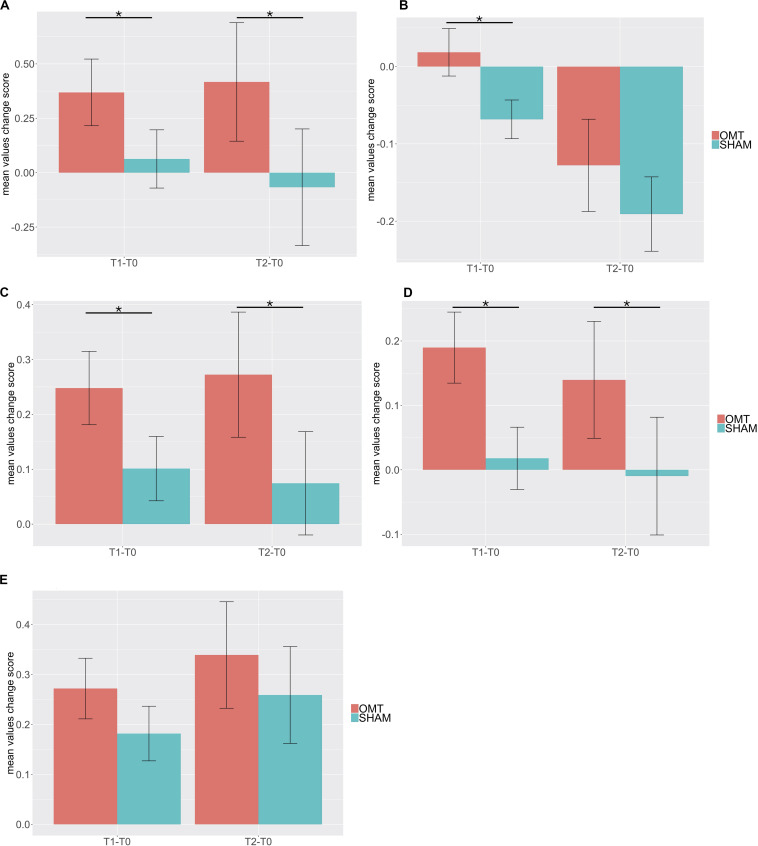
Bar chart of thermic baseline differences changes of different region of interest (ROI) during the Touch (T1–T0) and Post-Touch (T2–T0) period. Values are mean ± standard errors of the mean. **(A)** nose tip; **(B)** forehead; **(C)** left perioral; **(D)** right perioral; **(E)** chin. OMT, osteopathic manipulative treatment. Black lines indicate the significant effect of Group. *p < 0.01.

Multivariate analysis showed a significant increase on the OMT group as compared to the SHAM for the thermographic data of the nose, left and right perioral as well as for the forehead region but not for the chin (Table 3). *Post hoc* Bonferroni corrected analysis revealed that the group receiving OMT showed a significant increase of temperature from baseline both in the touch and post-touch periods (all comparisons *p* < 0.01). Whereas, the group receiving SHAM treatment showed no significant change (Figure 3).

**TABLE 3:** Multivariate linear mixed effect analysis for thermic outcomes.

	Beta	95% CI	*p* < |*t*|	Cohen’s *d*
Nose	0.38	0.12–0.63	<0.01	0.72
Left perioral	0.17	0.06–0.27	<0.001	0.59
Right perioral	0.16	0.07–0.24	<0.001	0.52
Forehead	0.07	0.01–0.12	<0.01	0.42
Chin	0.08	-0.02 to 0.18	0.13	0.40

*Data showed the comparison between osteopathic manipulative treatment versus sham therapy. The beta showed the values of the group as well as of epoch factor.*

**FIGURE 4:**
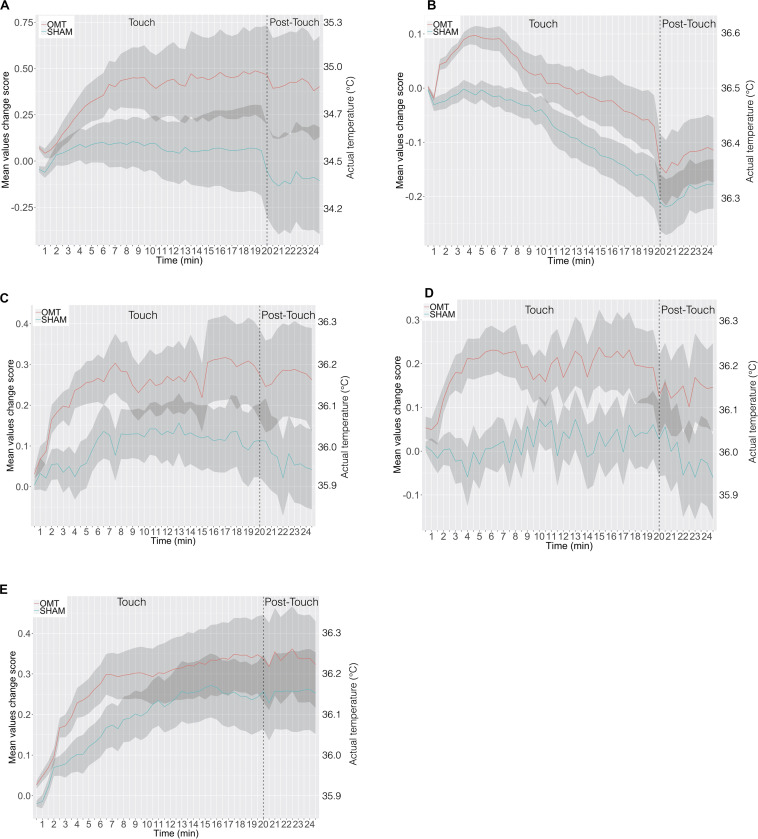
The time course in minutes of thermic data in response to osteopathic manipulative treatment and SHAM during the 20-min-long Touch & 5-min-long Post-Touch periods. **(A)** Nose tip; **(B)** forehead; **(C)** left perioral; **(D)** right perioral; **(E)** chin. Data are presented as change in temperature-per-minute from Baseline in each period. The shaded area represents ± 1 standard errors of the mean. OMT, osteopathic manipulative treatment.

To further explore this analysis, a trend analysis was performed looking at the differences compared to the baseline value of the touch and post-touch period. The OMT group showed a general faster and larger increase of temperature early in the touch period, which was sustained throughout the post-touch period, unless for the forehead, which showed a decrease. However, the SHAM group did not show this effect (Figure 4).

### Heart Rate Variability

**FIGURE 5:**
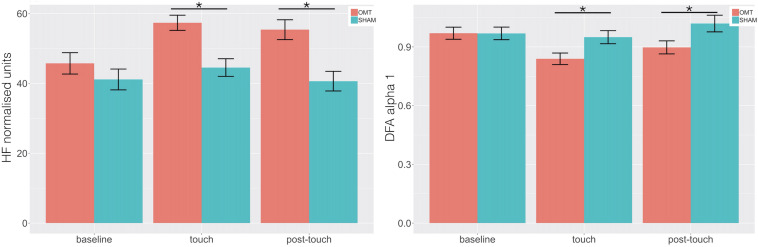
Bar chart displaying the mean heart rate variability (HRV) parameters values recorded in the baseline, Touch and Post-Touch period among the osteopathic and sham group. HF, high frequency in normalized unit; DFA-1, detrended fluctuation scaling exponent. Data presented are means ± standard errors of the mean. Black lines indicate the significant effect of Group. *p < 0.01. OMT, osteopathic manipulative treatment.

Multivariate analysis showed a statistically significant difference between OMT and SHAM groups on the nuHF (*p* < 0.001) (Figure 5). Bonferroni *post hoc* analysis showed that the OMT group significantly increased nuHF values compared to sham (*p* < 0.01).

Similarly, a statistically significant difference was revealed for DFA-a1 (*p* < 0.01). Bonferroni *post hoc* analysis demonstrated that the OMT group had a significant effect compared to SHAM (*p* < 0.01).

### Electrodermal Activity

**FIGURE 6:**
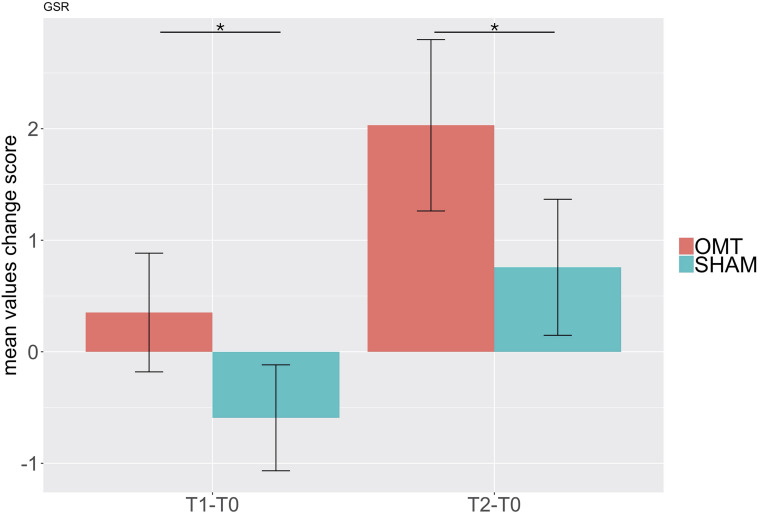
Bar chart of galvanic skin response (GSR) showing baseline differences changes during the Touch (T1–T0) and Post-Touch (T2–T0) period. Values are mean ± standard errors of the mean. Black lines indicate the significant effect of Group. *p < 0.01. OMT, osteopathic manipulative treatment.

Table 4 shows data for GSRs at different time points. Further MER analysis showed a statistically significant difference between the groups for the skin conductance values (1.09; 0.30–1.89; <0.01). Indeed, as demonstrated by Figure 6, participants who received the OMT significantly increased the GSR from baseline values both in touch and in post-touch periods. SHAM group did not show any significant change.

**TABLE 4:** Galvanic skin response (GSR) at different timepoints.

GSR	OMT	Sham	*p* > |*t*|
T0	1.06 ± 0.70	2.49 ± 0.53	0.10
T1	1.41 ± 0.81	1.90 ± 0.77	0.66
T2	3.09 ± 1.02	3.25 ± 0.74	0.90
Diff T1–T0	0.35 ± 0.76	−0.79 ± 0.60	<0.01
Diff T2–T0	2.03 ± 0.86	0.56 ± 0.64	<0.01

*Data are presented as mean ± standard error. *p* values from student *t* test.*

## Discussion

The present randomized cross-over study showed that the OMT increased the temperature on critical areas of the face, more specifically the nose, bilateral periorbital regions and forehead. In contrast, when healthy non-symptomatic adult volunteers received the sham therapy, which would respond to a gentle static touch, no significant change in the temperature was shown.

Osteopathic manipulative treatment was also associated with a specific variation of HRV parameters, that is an increase of HF domain and a reduction of DFA-a1 values, which was not seen when participants received sham treatment.

Moreover, osteopathy was shown to be associated with an increase of skin conductance, that means a sympathetic response, which did not appear on the sham group.

Part of these findings is consistent with our previous studies in adults, which report that a single osteopathic session produced a parasympathetic response to a significantly greater extent than sham or no-touch procedure (Ruffini et al., 2015).

The present study extends this previous work by investigating effects over different autonomic outputs and a longer study period. While previous research used only HRV measurements with changes in heart-rate, RR interval, over the treatment period, here we reported that not only the HRV parameters change but that this variation is also associated to a change in the temperature over specific face regions. Indeed, by analyzing high resolution thermal IRI to investigate the autonomic reactivity reflected in changes of facial temperature, we showed that when participants are exposed to osteopathic treatment, there is a significant increase of the temperature in specific areas of the face that are recognized being proxies of the ANS activity (Ioannou et al., 2014). For example, the tip of the nose has been used mainly as an indicator of autonomic response. Some research showed that nasal skin temperature might reflect an increase or decrease of arousal states, producing respectively vasoconstriction/temperature drop and vasodilatation/temperature raise, in different clinical and laboratory conditions (Mizukami et al., 1987; Tanaka et al., 1998; Nakayama et al., 2005; Nakanishi and Imai-Matsumura, 2008; Nozawa and Tacano, 2009; Kuraoka and Nakamura, 2011). Considering, instead, the other facial regions, increased forehead temperature may indicate activation of the corrugator muscle, which is highly correlated with mental stress (Ebisch et al., 2012; Zhou et al., 2013); consequently, a deactivation reflects mental relaxation. Conversely, perioral region thermal distribution is strictly related to the sudomotor response activity, given the high concentration of sweat glands in this area (Cardone and Merla, 2017). A decrease in the temperature over the perioral region would imply activation of the sweating function and consequently a reaction, that is directly linked to the sympathetic ANS.

We interpret the accompanying increase of these temperatures as reflecting the positive parasympathetic effect of osteopathic treatment on participants’ ANS. Considering the latencies and amplitudes of responses to OMT, we suggest that the faster and higher temperature increase reveals autonomic adjustments typically associated with parasympathetic, more specifically, vagal response. It is worth to note that induced parasympathetic vasodilatation reaction has many adaptive functions in the context of the “rest-digest” response, in particular, that of redistributing blood flow. This reaction generally corresponds to an increase in gastrointestinal tract irrigation, a reduction of the heart rate, activating the vagal nerve favoring, therefore, a calmer, more relaxed, recovery state response of the body. Another consequence of this reaction is that the face becomes red, which may signal an emotional state indicating the presence of a safe, relaxing environment.

Our findings are also consistent with recent studies indicating that the use of manual treatment can produce warmth in given areas treated (Walchli et al., 2014). Here we show these differential responses to OMT and sham on validated and standardized thermic areas using a robust methodological study design. However, analyzing closer the data of Walchli and colleagues showed that the mean temperature did not change between the manual procedures. Instead, higher variability in the variance of the sample, as well as the fact that the study was underpowered, might explain the group differences (Walchli et al., 2014). Thus, further research is required to confirm this data on autonomic functions and match with other measurements.

Other measures used in the present study were identified in previous recent research that determined the association between thermal data and the EDA, specifically GSR data (Vetrugno et al., 2003). Electrodermal activity has been demonstrated to reflect sympathetic cholinergic sudomotor function, a modification of which generates skin resistance changes and therefore electrical conduction alteration. Several studies demonstrated its validity, and therefore EDA has been recognized as a valid index of sudomotor function (Knezevic and Bajada, 1985a, b) as well as a sensitive index of bodily arousal (Boucsein et al., 2012). Galvanic skin response, which assesses EDA, indicates, therefore, a potential variation in the secretory activity of sweat glands that is independent of the vascular reaction.

Considering the limitations, the present study examined only the acute effects of a single osteopathic manipulative intervention on short-term measures of autonomic functions; in other words, a single session may have no predicted value compared to multiple sessions. The sustained effect of very short treatments is still unknown. Besides, we used pulse transducer data to calculate HRV, rather than the more common approach of using ECG. This measurement might limit direct comparison with studies that use ECG. Moreover, data acquisition using pulse transducers may be suboptimal because these devices might not accurately discriminate between the sinus and non-sinus beats. Furthermore, we enrolled healthy volunteers, who might react differently from patients. Indeed, patients with a low parasympathetic tone may see an effect, whereas healthy volunteers may see no effect. Finally, we did not use a pre-defined treatment protocol, in order to show an association between technique and outcomes, but we decided to mimic the routine osteopathic clinical practice where no rigid pre-defined protocols are applied.

Future work is needed to determine whether the use of IRI is suitable for clinical trials enrolling patients with different pathologies that have been demonstrated to benefit from OMT and whether thermal data might have a prognostic long-term, clinically significant, role. In conclusion, OMT is known to have a significant impact on different age groups (Channell et al., 2016; Ruffini et al., 2016; Lanaro et al., 2017). While osteopathy can have some benefits for diverse clinical conditions (Franke et al., 2014; Cicchitti et al., 2015; Bagagiolo et al., 2016; Racca et al., 2017; Arienti et al., 2018), a better understanding of the neurophysiological mechanisms underpinning these effects is required to improve protocols and plan trials targeting specific clinical conditions that can benefit the most. The present research supports the hypothesis that a single session of OMT as compared to sham induces sympathovagal autonomic effects in healthy non-symptomatic adults. Offering, therefore, insights for the development and further plan of manual therapies studies in the ANS context.

## Data Availability Statement

The datasets generated for this study are available on request to the corresponding author.

## Ethics Statement

The studies involving human participants were reviewed and approved by Institutional Ethics Committee of the University “G. D’Annunzio” of Chieti-Pescara. Written informed consent was obtained from the individual for the publication of any potentially identifiable images or data included in this article.

## Author Contributions

AP, FS, and AM supervised the experiment and reviewed the manuscript for intellectual content. DC exported the data and reviewed the manuscript. FC ran the statistical analysis, supervised the research and reviewed the manuscript for intellectual content. All authors conceived the idea, drafted and approved the final version of the manuscript.

## Conflict of Interest

The authors declare that the research was conducted in the absence of any commercial or financial relationships that could be construed as a potential conflict of interest.

## Footnotes

1 http://kubios.uef.fi
